# Longitudinal genome-wide DNA methylation analysis uncovers persistent early-life DNA methylation changes

**DOI:** 10.1186/s12967-018-1751-9

**Published:** 2019-01-09

**Authors:** Raúl F. Pérez, Pablo Santamarina, Juan Ramón Tejedor, Rocío G. Urdinguio, Julio Álvarez-Pitti, Pau Redon, Agustín F. Fernández, Mario F. Fraga, Empar Lurbe

**Affiliations:** 1 Cancer Epigenetics Laboratory, Institute of Oncology of Asturias (IUOPA)-Instituto de Investigación Sanitaria del Principado de Asturias (ISPA)-Hospital Universitario Central de Asturias (HUCA), 33011 Oviedo, Asturias Spain; 2 Nanomedicine Group, Nanomaterials and Nanotechnology Research Center (CINN-CSIC), Universidad de Oviedo, 33940 Oviedo, Asturias Spain; 3 Servicio de Pediatría, Consorcio Hospital General Universitario de Valencia, 46014 Valencia, Spain; 4 Centros de Investigación Biomédica en Red de Fisiopatología Obesidad y Nutrición (CB06/03), Instituto de Salud Carlos III, Madrid, Spain

**Keywords:** Epigenetics, DNA methylation, Histone modification, Aging, Newborn, Longitudinal

## Abstract

**Background:**

Early life is a period of drastic epigenetic remodeling in which the epigenome is especially sensitive to extrinsic and intrinsic influence. However, the epigenome-wide dynamics of the DNA methylation changes that occur during this period have not been sufficiently characterized in longitudinal studies.

**Methods:**

To this end, we studied the DNA methylation status of more than 750,000 CpG sites using Illumina MethylationEPIC arrays on 33 paired blood samples from 11 subjects at birth and at 5 and 10 years of age, then characterized the chromatin context associated with these loci by integrating our data with histone, chromatin-state and enhancer-element external datasets, and, finally, validated our results through bisulfite pyrosequencing in two independent longitudinal cohorts of 18 additional subjects.

**Results:**

We found abundant DNA methylation changes (110,726 CpG sites) during the first lustrum of life, while far fewer alterations were observed in the subsequent 5 years (460 CpG sites). However, our analysis revealed persistent DNA methylation changes at 240 CpG sites, indicating that there are genomic locations of considerable epigenetic change beyond immediate birth. The chromatin context of hypermethylation changes was associated with repressive genomic locations and genes with developmental and cell signaling functions, while hypomethylation changes were linked to enhancer regions and genes with immunological and mRNA and protein metabolism functions. Significantly, our results show that genes that suffer simultaneous hyper- and hypomethylation are functionally distinct from exclusively hyper- or hypomethylated genes, and that enhancer-associated methylation is different in hyper- and hypomethylation scenarios, with hypomethylation being more associated to epigenetic changes at blood tissue-specific enhancer elements.

**Conclusions:**

These data show that epigenetic remodeling is dramatically reduced after the first 5 years of life. However, there are certain *loci* which continue to manifest DNA methylation changes, pointing towards a possible functionality beyond early development. Furthermore, our results deepen the understanding of the genomic context associated to hyper- or hypomethylation alterations during time, suggesting that hypomethylation of blood tissue-specific enhancer elements could be of importance in the establishment of functional states in blood tissue during early-life.

**Electronic supplementary material:**

The online version of this article (10.1186/s12967-018-1751-9) contains supplementary material, which is available to authorized users.

## Background

Epigenetic modifications such as DNA methylation are known to influence gene expression and thus biological function [1] and alterations in epigenetic marks are found in processes ranging from physiological development and cellular differentiation [2] to pathological scenarios, as well as aging [3, 4]. Reflecting the developmental changes of the individual, most epigenetic changes occur during embryogenesis, where waves of extensive epigenetic reprogramming take place [5]. After birth, epigenetic modification continues to take place throughout the human lifespan, both at the DNA methylation and chromatin levels [6] and precise DNA methylation markers of age have recently been developed [7].

It seems, however, that the first years of life constitute the post-natal period with the most substantial epigenetic changes [8, 9] and, moreover, prenatal and early life epigenetic aggression has been linked to countless health-related consequences in a wide variety of settings [10]. Thus, the characterization of childhood DNA methylation changes could help uncover genomic locations of functional relevance.

While age-related changes have been functionally linked to many biological processes, other alterations are known to arise in a stochastic fashion [11, 12]. As such, the use of longitudinal experimental designs can allow for the identification of DNA methylation alterations in a more controlled environment [9, 13–16], and this approach has also been employed to study the influence of clinical parameters on the epigenome, especially during early childhood [17]. However, longitudinal DNA methylation studies which analyze data from more than two time points remain scarce [17–19] and most of the previous literature stands on now surpassed methylation screening technologies such as the Infinium Human Methylation 450K BeadChip which mainly interrogates CpG-dense genomic regions, even though the functional association between DNA methylation and gene expression is increasingly being described for CpG-sparse locations such as enhancers and gene bodies [20].

This study, therefore, presents, to the best of our knowledge, the first 3-point longitudinal genome-wide DNA methylation analysis of blood tissue employing the Illumina Infinium MethylationEPIC BeadChip, which allowed us to characterize two distinct phases of early-life epigenetic changes during the first 10 years of life at the same time as focusing on various types of genomic regions, using a clinically well-characterized cohort. Furthermore, we integrated our generated data with external chromatin and enhancer datasets in order to obtain a functional view of the observed age-related DNA methylation changes. Finally, we applied bisulfite pyrosequencing in order to technically and biologically validate the results obtained in the discovery and independent cohorts, respectively.

## Methods

### Selection of participants

Newborn children born at term (gestational age ≥ 37 weeks) after uncomplicated pregnancies and in the absence of perinatal illness in the General Hospital, University of Valencia, Spain, were randomly selected to enroll in the study. The characteristics of each gestation and delivery were obtained from routine obstetrical records, and the gestational age at birth was measured using the method of Ballard et al. [21]. Groups were established on the basis of birth weight: < 10th percentile for their sex (SGA); between 10th and 90th percentile (AGA); and > 90th percentile (LGA) [22]. The subjects were followed-up at 5 and 10 years of age.

### Anthropometric parameters and biochemical analyses

At both 5 and 10 years, height was measured to the nearest 0.5 cm using a standardized wall-mounted height board, and body weight to the nearest 0.1 kg using a standard beam balance scale with the barefoot subjects wearing light clothing. Body mass index (BMI) was calculated as the weight in kilograms divided by the square of the height in meters (see Table 1 and Additional file 1: Table S9 for this and other clinical information).

**Table 1:** Clinical characteristics of the 11 subjects enrolled in the study

Subject At 0 years	Sex	Birth	Lactation	Gestational age (weeks)	Cephalic perimeter (cm)	Birth weight (g)	Birth weight (group)	Birth length (cm)
1	Male	VD	FF	40	35.0	3580	AGA	51.0
2	Male	CS	FF	40	34.0	3800	AGA	50.0
3	Male	VD	FF	38	31.0	2155	SGA	46.0
4	Male	VD	FF	37	32.5	2720	AGA	47.0
5	Female	VD	BF	37	33.0	2540	AGA	47.0
6	Female	VD	BF	38	32.0	2400	AGA	48.5
7	Male	CS	FF	41	35.0	3550	AGA	52.5
8	Female	CS	FF	38	34.5	3540	AGA	49.5
9	Female	VD	BF	39	33.0	3940	LGA	51.0
10	Male	VD	FF	39	35.0	3860	LGA	50.0
11	Female	CS	BF	39	31.0	2340	SGA	45.5

Subject At 5 years	Creatinine	Insulin	HDL	LDL	Triglycerides	Weight (g)	Body mass index	Height (cm)
1	0.3	1.8	47	101	60	18,700	14.6	113.0
2	0.4	2.0	60	123	93	22,700	21.4	103.0
3	0.6	2.9	52	118	55	17,000	13.8	111.0
4	0.3	5.8	61	120	69	15,700	14.9	102.5
5	0.3	4.7	58	103	79	22,500	16.7	116.0
6	0.3	2.7	56	109	56	16,600	14.4	107.5
7	0.3	2.1	44	109	45	16,800	14.1	109.0
8	0.4	7.0	59	98	69	26,100	18.6	118.5
9	0.3	4.5	47	118	81	22,000	15.2	120.5
10	0.4	4.2	83	128	58	22,500	17.0	115.0
11	0.4	7.6	54	66	34	23,100	14.6	113.0

Subject At 10 years	Creatinine	Insulin	HDL	LDL	Triglycerides	Weight (g)	Body mass index	Height (cm)
1	0.5	3.3	47	110	52	27,300	15.6	132.5
2	0.5	3.7	53	117	69	37,600	22.9	128.0
3	0.6	5.2	59	122	46	31,500	14.6	147.0
4	0.5	10.9	64	134	59	34,100	17.2	141.0
5	0.5	12.5	55	147	77	42,900	21.3	142.0
6	0.4	9.1	48	97	66	30,000	15.8	138.0
7	0.4	7.1	40	92	64	27,800	16.5	129.8
8	0.5	9.7	61	111	52	46,000	21.5	146.2
9	0.4	3.2	47	88	88	30,100	17.7	130.5
10	0.6	4.5	99	107	41	38,200	18.3	144.5
11	0.6	6.3	52	65	33	37,000	17.6	145.0

*FF* formula feeding, *BF* breast feeding, *VD* vaginal delivery, *CS* cesarean section, *AGA* appropriate for gestational age, *SGA* small for gestational age, *LGA* large for gestational age, *HDL* high-density lipoprotein, *LDL* low-density lipoprotein

### Sample collection, DNA extraction, and quantification

Blood samples were collected from a total of 29 subjects at different testing times (n = 51). Cord blood samples were taken at birth and peripheral venous blood samples were taken from each child during their fifth and tenth year of life. Genomic DNA was extracted with the RealPure kit (RealPure, REAL, Durviz) and quantified with the Nanodrop-2000C Spectrophotometer. A DNA quality check was performed with Quant-iT PicoGreen dsDNA reagent.

### Genome-wide DNA methylation analysis

Microarray-based DNA methylation profiling was performed with the Illumina Infinium MethylationEPIC BeadChip on 33 paired blood samples from 11 subjects collected at birth, 5 and 10 years of age. Bisulfite conversion of DNA was performed using the EZ DNA methylation kit (Zymo Research) following the manufacturer’s recommendations, but with the modifications described in the Infinium assay methylation protocol guide. Processed DNA samples were then hybridized to the BeadChip following the Illumina Infinium HD methylation protocol at the Centro Nacional de Genotipado (CEGEN-ISCIII, Spain, http://www.cegen.org).

### DNA pyrosequencing

The DNA methylation status of representative CpG sites (cg07547765, cg11047325, cg03830443) was evaluated by bisulfite pyrosequencing using the primers described in Additional file 2: Table S8. Technical validations were performed for all 11 subject samples at the three time points (n = 33) and biological validations were performed on two independent cohorts: (1) peripheral blood from 9 children at birth and 5 years of age (n = 18), and (2) peripheral blood from 9 different children at 5 and 10 years (n = 18). Genomic DNA was isolated following standard phenol–chloroform extraction protocols. Bisulfite conversion of isolated DNA was performed in accordance with the EZ DNA methylation-gold kit (Zymo Research) following the manufacturer’s instructions. After PCR amplification, pyrosequencing was performed using PyroMark Q24 reagents and a vacuum prep workstation, equipment and software (Qiagen).

### MethylationEPIC BeadChip data preprocessing

All analyses were carried out using the statistical software R (version 3.4.2). IDAT files from the methylationEPIC BeadChip were preprocessed following a pipeline based on the R/Bioconductor package *minfi* (version 1.22.1) [23]. Probes where at least one sample had a detection p-value > 0.01, probes located in chromosomes X and Y, probes overlapping genetic variants [24] and crossreactive and multimapping probes [25] were discarded for downstream analyses. β-values were normalized using the Noob method [26] implemented in *minfi*, followed by a BMIQ normalization [27] implemented in the R/Bioconductor package *ChAMP* (version 2.8.9) [28]. M-values were calculated from the normalized β-values through a logit transformation (R/Bioconductor package *lumi*, version 2.28.0) [29] and employed for downstream analyses. Multidimensional scaling (MDS) and principal component regression analyses were used to identify potential confounding variables (R/Bioconductor packages *Enmix*, version 1.12.4) [30]. When specified, blood cell-type composition was calculated by the Houseman method [31]. However, to correct for batch effects and unwanted sources of variation in the data we performed a surrogate variable analysis [32] with the R/Bioconductor package *sva* (version 3.23.4) [33] because this procedure seeks to adjust for any confounders, including cell-type heterogeneity [34]. The number of latent factors was estimated using the “*leek*” method, but it is of note that no surrogate variables were found after the normalization procedures for our generated datasets.

### Differential DNA methylation analyses

Differentially methylated probes (dmCpGs) were calculated with the R/Bioconductor package *limma* (version 3.32.10) [35]. A linear model was fitted between methylation levels as response variable, the variable of interest (*age* group), the aforementioned surrogate variables and the information of the paired samples (*patient* group) for each of the analyses: 0 vs. 5 and 5 vs. 10 years old. The set of p-values obtained from the tests was adjusted for multiple comparisons using the Benjamini–Hochberg method to control for false discovery rate (FDR < 0.05). An additional threshold of shift size was applied, filtering out significant probes with M-value changes of less than 0.5, as has been suggested elsewhere [36]. Venn diagram representations of the relationships between statistically significant dmCpGs were generated with the online resource provided by the UGent/VIB bioinformatics unit (available at http://bioinformatics.psb.ugent.be/webtools/Venn/). Further enrichment analyses were performed by means of two-sided Fisher’s tests (*p *< 0.05 significance threshold), measuring effect size either by odds ratio (OR), or by the difference between observed counts and expected hypergeometric means, employing appropriate backgrounds for the interrogated probes in each given context.

### Density of CpG analysis

For each of the probes in the methylation arrays, density of CpG was measured as the number of genomic CpGs present divided by the number of those possible in a 2 kbp window centered on the CpG location under study. Wilcoxon non-parametric tests were used to determine whether there were significant differences between the density distributions of the CpGs belonging to each subset of interest and the densities of the array probes in the background. A significance level of 0.05 was employed for all tests. Shift size was measured using median differences and Cliff’s Delta (CD).

### CGI status and genomic region analysis

CGI (CpG island) membership was assigned to each probe using the Illumina EPIC annotation with the R/Bioconductor package and *IlluminaHumanMethylationEPICanno.ilm10b2.hg19* (version 0.6.0). Genomic region position was assigned using the R/Bioconductor packages *TxDb.Hsapiens.UCSC.hg19.knownGene* (version 3.2.2) and *ChIPseeker* (version 1.12.1) [37]. Statistical significance with respect to concrete CGI status or genomic region was determined by two-sided Fisher’s tests (significance level *p *< 0.05), and ORs were used as a measure of the association effect with respect to a particular feature. Appropriate backgrounds which included all the probes interrogated by the EPIC array in each of the comparisons were used for statistical purposes.

### Region set enrichment analysis

Enrichment analyses were performed with the R/Bioconductor package *LOLA* (version 1.6.0) [38], which looks for over-enrichment by conducting one-sided Fisher’s tests (p-value < 0.05 significance threshold), by comparing overlap of probes (10 bp probe-centered windows) with the dataset of interest. Enrichment of histone marks was determined using histone ChIP-seq peak tracks (H3K4me1, H3K4me3, H3K27me3, H3K36me3, H3K9me3 and H3K27ac marks) from 98 epigenomes (primary tissues, cultures and cell lines) obtained from the NIH Roadmap and ENCODE projects [39, 40] and integrated into the *LOLA* extended software (datasets obtained from http://databio.org/regiondb). The same method was employed for chromatin-segment analysis using the expanded NIH Roadmap ChromHMM 18-state model tracks for the same 98 epigenomes, generated from the previous histone marks (custom database generated with data obtained from http://egg2.wustl.edu/roadmap/). In a similar fashion, enhancer enrichment analysis was performed using the enhancer tracks defined in the EnhancerAtlas database [41] (custom database generated with data obtained from http://enhanceratlas.org/download.php) for 65 genomes (primary tissues, cultures and cell lines) defined by the consensus combination of different independent experimental datasets such as histone marks, DNAse hypersensitive sites (DHS) and transcription factor binding sites (TFBS). This last dataset was also used to create the CpG-gene network shown in Fig. 4c: CpGs were first mapped to enhancers in at least one of the 22 EnhancerAtlas tracks corresponding to blood tissue and cell type, and afterwards plotted using the R/CRAN package *igraph*, where network nodes represent CpG sites or genes, while network edges reflect interactions between the enhancer element containing the CpG and the corresponding associated gene.

### Gene ontology analyses

Gene ontology enrichments were calculated using the R/Bioconductor package *missMethyl* (version 1.10.0 *gometh* function) [42], which performs one-sided hypergeometric tests taking into account and correcting for any bias derived from the use of differing numbers of probes per gene interrogated by the array. The annotation database that was interrogated is contained within the R/Bioconductor package *GO.db* (version 3.4.1). Appropriate backgrounds of total probes for each given context were employed in the corresponding analyses. Additionally, because a great number of genes contained both hyper- and hypomethylated probes, certain gene ontology enrichments were performed only on those genes that were exclusively hyper- or hypomethylated. Further analyses were also performed by grouping the annotated CpGs into gene regions, using the annotations provided in Additional file 3: Table S1 (column “annotation”): “promoter” is formed by collapsing “Distal promoter” and “Promoter (≤ 1 kb)”, “gene body” is formed by collapsing “3′ UTR”, “5′ UTR”, “Intron”, “Exon” and “Downstream”). epigenomes Additional file 4: Table S6 contains the full information on the gene ontology analyses. To help with the visualization of the results, the ontologies were graphically summarized using the online tool REViGO (available at http://revigo.irb.hr/) [43], which performs semantic similarity analyses in order to reduce the number of redundant functional terms obtained.

## Results

### Genome-wide profiling of DNA methylation changes during the first 10 years of life

**Fig. 1:**
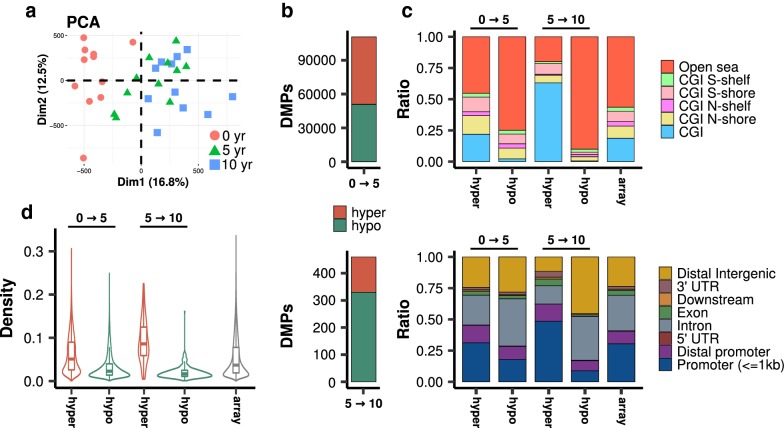
DNA methylation changes during the first years of life. **a** Principal Component Analysis (PCA) for 783659 CpG sites across all samples in our study. The two PCs represented explain approximately 30% of the variance in methylation levels. **b** Stacked barplots indicating the total number of dmCpGs detected as either 0 → 5 or 5 → 10 changes (see Additional file 3: Table S1 for the detailed names and annotations of the dmCpGs). **c** Stacked barplots depicting the relative distribution of dmCpGs and of the array background according to their CpG island-related or gene location-related status. **d** Violin plots showing the distribution of genomic CpG density for the detected dmCpGs and array background

To characterize DNA methylation changes that occur during the first years of life, we analyzed the methylation status of 783,659 CpG sites in cord, 5-year-old and 10-year-old paired blood samples from 11 subjects (see Table 1 and Fig. 1a) using the Illumina Infinium MethylationEPIC BeadChip. After preprocessing our data (see “Methods”), we applied an empirical Bayes moderated t test to identify CpGs differentially methylated between the different age groups (dmCpGs; FDR < 0.05) (see “Methods”). We found 110,726 CpGs whose methylation level significantly changed from birth to 5 years (0 → 5), but only 460 from 5 to 10 years (5 → 10). The 0 → 5 widespread methylation changes consisted of, respectively, 59,749 and 50,977 CpGs hyper- or hypomethylated over time, while 130 and 330 CpGs, respectively, were found to be hyper- or hypomethylated over the 5 → 10 years period (Fig. 1b, Additional file 3: Table S1). Notably, hypomethylation changes were found to be more pronounced than hypermethylation changes between 0 and 5 years, while the magnitude of change was more balanced in both directions between 5 and 10 years (Additional file 5: Figure S1).

Next, we determined the genomic distribution of the dmCpGs identified, distinguishing between CpG island-related regions and gene-related regions (Fig. 1c). As compared to the distribution of the array background, hypermethylated CpGs were found to be enriched at CpG islands for both 0 → 5 and 5 → 10 changes (Fisher’s test; both p < 0.001, odds ratios (ORs) = 1.2 and 7.4, respectively) and impoverished at open sea locations (Fisher’s test; both p < 0.001, ORs = 0.6 and 0.2 respectively), while the inverse was the case for hypomethylated CpGs, that is they were very enriched at open sea regions (Fisher’s test; both p < 0.001, ORs = 2.4 and 6.9, for 0 → 5 and 5 → 10, respectively) and very impoverished at CpG islands (Fisher’s test; both p < 0.001, ORs = 0.1 and 0.03 for 0 → 5 and 5 → 10, respectively). As regards gene-related locations, hypermethylated CpGs were enriched at promoters and distal promoters (Fisher’s test; both p < 0.001, ORs = 1.04 and 1.5 for 0 → 5 and both p < 0.001, ORs = 2.2 and 1.4 for 5 → 10) and impoverished at intronic regions for 0 → 5 changes (Fisher’s test; p < 0.001, OR = 0.8) and at both intronic and intergenic regions for 5 → 10 changes (Fisher’s test; both p < 0.001, ORs = 0.4 and 0.4), while in contrast, hypomethylated CpGs were preferentially found at intronic and intergenic regions for both age bands, (Fisher’s test; p < 0.001, ORs = 1.6 and 1.3, in these regions respectively, for 0 → 5 and p < 0.01 and < 0.001, ORs = 1.4 and 2.7, respectively, for 5 → 10).

Lastly, we analyzed the genomic density of the dmCpGs found (see “Methods”), which confirmed that hypermethylation occurred at genomic regions dense in CpG dinucleotides, while hypomethylation appeared at CpG-poor regions (Fig. 1d, Wilcoxon test; all p < 0.001, median differences (MD) and Cliff’s deltas (CD) compared to array background 0.014/0.15 and − 0.014/− 0.29 for 0 → 5 and 0.05/0.5 and − 0.02/− 0.5 for 5 → 10 hyper- and hypomethylated CpGs, respectively).

**Fig. 2:**
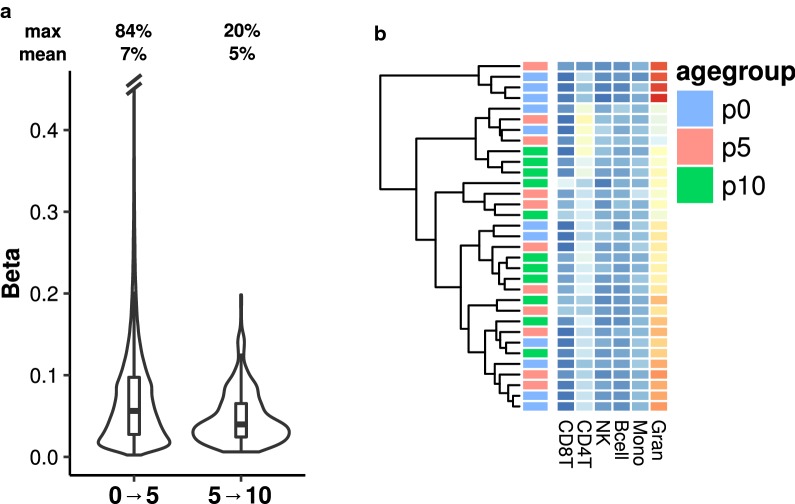
DNA methylation changes and composition of cell types. **a** Violin plots indicating the distribution of absolute changes in β-value for 0 → 5 and 5 → 10 dmCpGs. **b** Heatmap depicting the different predicted proportions of blood cell-types for the different samples, which are clustered by unsupervised hierarchical clustering

On the whole, these analyses reveal strong differences between DNA methylation changes that occur during the first years of life, where extensive methylation changes are observed between 0 and 5 years, while only moderate changes occur between 5 and 10 years. Strikingly, while the genomic distribution of these changes appeared to be similar, we found that the changes occurring in the first lustrum of life were of a greater magnitude that those occurring in the second lustrum (Fig. 2a, Wilcoxon test; p < 0.001, MD = 0.02, CD = 0.2). Moreover, these differences, which were predicted by the PCA analysis (Fig. 1a), did not seem to be related to any major changes in the composition of blood cell types, as shown by the hierarchical clustering analysis of their predicted cell types (see “Methods” and Additional file 6: Table S2), where the samples did not show extensive age-associated clustering (Fig. 2b).

### Chromatin and functional context of early-years DNA methylation changes

In order to study the functional context in which the DNA methylation changes identified occurred, we integrated our results with previously published data from NIH Roadmap Epigenomics and ENCODE relating to ChIP-seq experiments describing the genome-wide localization of the histone modifications H3K4me1, H3K4me3, H3K27ac, H3K36me3, H3K27me3 and H3K9me3 across different blood cell types and cell lines (see “Methods”). Due to the low number of probes, most of the enrichment analyses were unsuccessful for 5 → 10 changes (results available in the Additional file 7: Table S3, Additional file 8: Table S4, Additional file 9: Table S5).

**Fig. 3:**
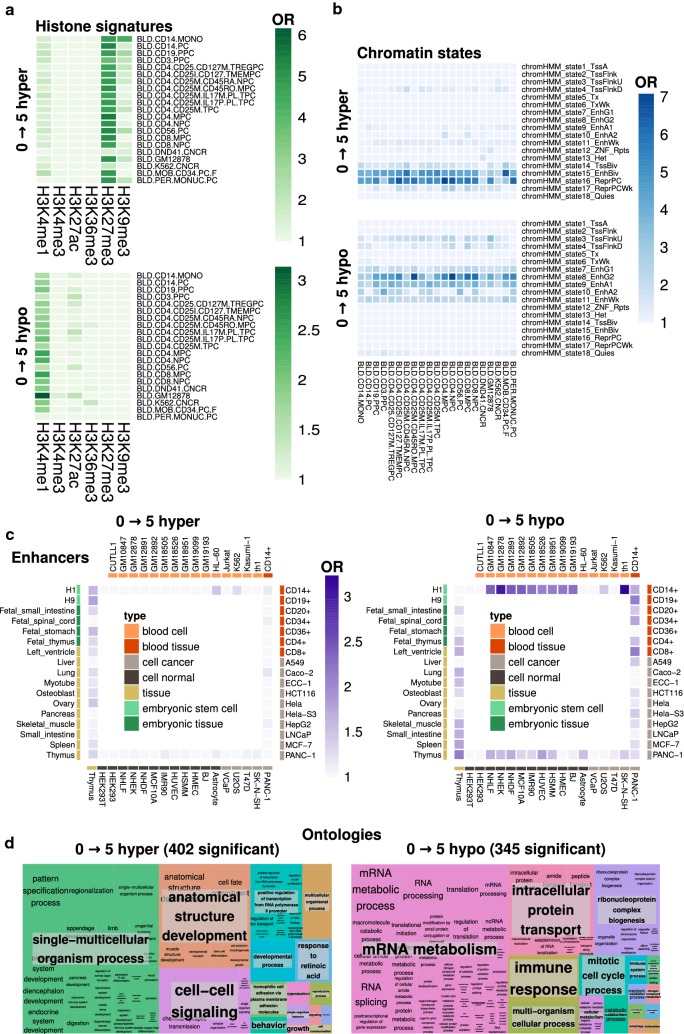
Chromatin and functional context of DNA methylation changes. **a** Heatmaps depicting significant (p < 0.05) enrichment of hyper- and hypomethylated 0 → 5 dmCpG sites with different histone marks, in a selection of 21 different blood tissue and cell types (see Additional file 7: Table S3 for 98 full cell and tissue types, and also 5 → 10 dmCpG enrichments). The color code indicates the significant enrichment based on the odds ratio (OR). **b** Heatmaps displaying significant (p < 0.05) enrichment of hyper- and hypomethylated 0 → 5 dmCpG sites with different chromatin states, in a selection of 21 different blood tissue and cell types (see Additional file 8: Table S4 for 98 full cell and tissue types, and also 5 → 10 dmCpG enrichments). The color code indicates the significant enrichment based on the OR. **c** Heatmaps showing significant (p < 0.05) enrichment of hyper- and hypomethylated 0 → 5 dmCpG sites with predicted enhancers in 65 different cell and tissue types (see Additional file 9: Table S5 for full data, and also 5 → 10 dmCpG enrichments). The color code indicates the significant enrichment based on the OR. **d** Treemap plots indicating the results of REViGO semantic analyses of significantly enriched (FDR < 0.05) gene ontology biological process terms for genes containing 0 → 5 dmCpGs. In total, 402 and 345 significant terms were found for hyper- and hypomethylated dmCpGs, respectively (see Additional file 4: Table S6 for full results, including molecular function and cellular component terms, and also 5 → 10 dmCpG enrichments)

By using this approach, we found that CpGs that were hypermethylated during the first 5 years of life were strongly associated with the repressive histone modification H3K27me3, while hypomethylated CpGs were associated with the active histone modification H3K4me1 (Fig. 3a, see Additional file 7: Table S3 for the full data).

Subsequently we analyzed the enrichment of these dmCpGs for 18 chromatin states, as defined in a Hidden Markov Model (HMM) built from the previous histone modifications (see “Methods”). The results evidenced that hypermethylation occurred at enhancer states associated with bivalent chromatin (i.e. formed by the simultaneous presence of repressive and active marks) and polycomb repressive domains, while hypomethylation occurred at states associated with enhancers, especially of the intragenic type (Fig. 3b, see Additional file 8: Table S4 for the full data). To gain a deeper understanding of enhancer-associated methylation changes, we integrated our data with the EnhancerAtlas database of predicted enhancers across 65 tissues (see “Methods”). This approach enabled us to characterize hypermethylated CpGs as being weakly associated with enhancers present in tissue and embryonic tissue whereas hypomethylated CpGs appeared more strongly associated with enhancers across all datasets, but particularly those specifically related to blood tissue (blood cell lines and primary cells) (Fig. 3c, see Additional file 9: Table S5 for the full data). Taken together, these results highlight the different functionality of the regions where DNA methylation is gained or lost during the first 5 years of life and suggest that hypomethylation could be more relevant than hypermethylation in the establishment of epigenetic patterns at enhancers controlling tissue-specific functions.

Lastly, we performed gene ontology analyses on the genes containing the dmCpGs. In order to avoid bias due to genes with both hyper- and hypomethylated dmCpGs, we conducted the analyses on those genes that were either exclusively hypermethylated or exclusively hypomethylated, also controlling for differences in the number of CpGs analyzed for each gene in the array (see “Methods”). This led to the finding that hypermethylated genes were mainly linked to developmental and cell-to-cell signaling processes, albeit with other notable ontologies also involved, such as response to retinoic acid. Hypomethylated genes, on the other hand, were enriched in metabolic terms related to mRNA and protein metabolism, immune response and mitosis (Fig. 3d, see Additional file 4: Table S6 for the full data). Curiously, when looking at the ontologies of dmCpGs belonging to genes that experienced both hyper- and hypomethylation, the main roles of the genes affected were with respect to cell surface signaling and intracellular component transport (Additional file 10: Figure S2A, Additional file 4: Table S6). This suggests that genes undergoing both hyper- and hypomethylation may well have different cellular functions than those that are differentially methylated in one direction only. This prompted us to examine the ontologies for all of the hyper- or hypomethylated probes (i.e. without taking into account the fact that they might be associated with genes that also contained probes with change in the opposite direction), which were also different (Additional file 10: Figure S2B, Additional file 4: Table S6): while the 0 → 5 hypermethylation ontologies did not substantially change, the hypomethylation terms appeared related more to cellular function, localization and activation, and membrane function, with immune terms being were maintained (Additional file 10: Figure S2, Additional file 4: Table S6). This last observation implies that not taking into account the existence of simultaneously hyper- and hypomethylated genes could alter the results obtained by ontology-enrichment analyses.

Taking into account these last findings, we extended our analyses to characterize gene ontologies depending on the gene region of the associated dmCpGs. We divided the 0 → 5 dmCpGs into promoter-, exon-, intron- or gene body-associated, hyper- or hypomethylated dmCpGs (8 groups in total), in order to identify differences, for example, between the ontologies of genes that suffered DNA methylation changes in their promoters versus genes with changes in their gene bodies. The results (Additional file 11: Figure S3, Additional file 4: Table S6), when compared to those previously found without segregating dmCpGs by context (shown in Fig. 3d) showed that genes associated to different functions suffered changes at different locations. For hypermethylated CpGs (Additional file 11: Figure S3A), the main functions related to development and signaling seen in Fig. 3d were found on genes with promoter- and exon-associated DNA methylation gains, while the functions associated to genes containing exon- or gene body-(the majority of which is exons) methylation increases were considerably different, manifesting functions such as GTPase signaling, ion transport or cell projection. For hypomethylated CpGs (Additional file 11: Figure S3B), the initially observed functions related to mRNA metabolism, protein transport or immune response were also found in genes with promoter-associated DNA methylation loses (which were, though, much more enriched in immunological functions), while, again, the functions observed for genes containing exon- or gene body-associated methylation reduction changed, being more related to signal transduction and movement of subcellular components. On the whole, these observations reinforce the idea that DNA methylation changes occur at different gene regions depending on the functionality of the affected gene.

### Persistent DNA methylation changes during the first 10 years of life

**Fig. 4:**
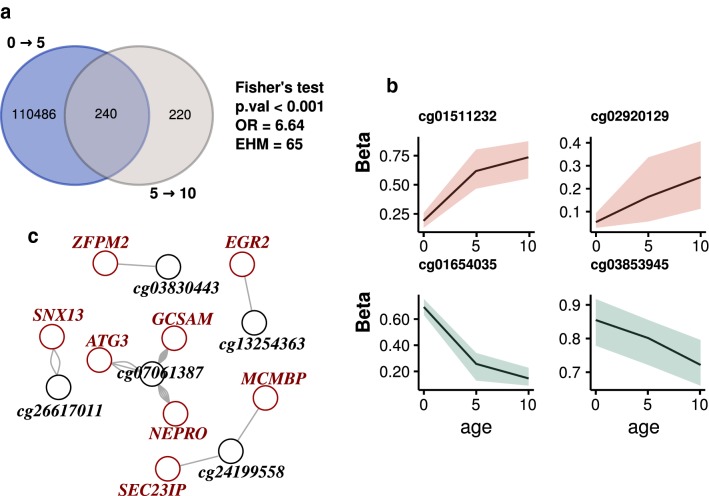
Comparison of 0 → 5 and 5 → 10 DNA methylation changes. **a** Venn diagrams illustrating the overlap between 0 → 5 and 5 → 10 dmCpGs (the results of a Fisher’s test for over-enrichment of the overlap is shown). **b** Plots illustrating the age-dependent change in β-value for 4 examples of dmCpGs common to both 0 → 5 and 5 → 10 (see Additional file 12: Figure S4 for all 36 CpG plots). **c** Network of gene–enhancer interactions observed between dmCpGs mapped to enhancer elements in blood tissue and cell types and associated genes. The number of connections between nodes reflects the mapping of a dmCpG to enhancers in more than one track

After conducting the analyses of DNA methylation, we made use of the longitudinal design of our study to compare the changes occurring in the 0 → 5 compared to the 5 → 10 period. Interestingly, we discovered an over-enriched subset of 240 dmCpGs that were altered in the first 5 years of life which continued to change in the following 5 years [Fig. 4a, Fisher’s test; p < 0.001, OR = 6.6, expected hypergeometric mean (EHM) = 65]. No significant gene ontologies were found for this set after correcting for multiple comparisons, although the top hypermethylation terms were related to developmental processes, and those for hypomethylation with metabolic and immunological activities (data not shown).

We subsequently filtered out CpGs with changes of less than 10% in β-value in order to retain those with more plausible biological roles, leaving 36 CpGs (Table 2). These remaining CpGs manifested two kinds of behavior: either strong first-lustrum changes with weaker second-lustrum changes, or a trend of constant (moderate) change (Fig. 4b, see Additional file 12: Figure S4 for all 36 CpG plots). A total of 16 different genes related to these select 0 → 5→10 dmCpGs, and, notably, two of the 36 CpGs were mapped to the HOXB7 gene. Since a proportion of these CpG sites were not associated with any gene and many of them were located in open sea regions (see Table 2), we made use of the EnhancerAtlas database to map them to enhancer elements in the 22 different types of blood tissue and cells shown in Fig. 3c (see “Methods”), and constructed a network with the linked to the enhancer elements containing the dmCpGs (Fig. 4c). In this way we thereby revealed additional relationships with other genes, with some sites, such as cg07061387, showing relationships with up to 3 different genes. Taken together these results indicate that the majority of the later-years epigenetic changes seem to be a weaker continuation of those that occur during the first 5 years of life, although there also appear to be certain CpG sites that show a more linear, and constant, trend of change along the first 10 years of life.

**Table 2:** CpG sites that change substantially during the first 10 years of life

Name	Location	UCSC RefGene	Name	Location	UCSC RefGene	Name	Location	UCSC RefGene
cg13949829	Island	MIR1225;PKD1	cg12024906	Island	HKR1	cg07690222	Open Sea	TMTC2
cg01323777	Island	KCNAB3	cg23201812	Open Sea		cg05498680	Open Sea	
cg00002033	Island	LRFN1	cg01654035	Open Sea		cg11047325	Island	SOCS3
cg23669081	Island	HOXB7	cg03853945	Open Sea		cg01331772	Open Sea	
cg17238334	Open Sea	LOC102477328	cg18311495	Open Sea		cg16022195	S-Shore	
cg01511232	Island		cg26617011	Open Sea		cg03465600	Open Sea	PARD3B
cg01577707	Open Sea	MIR100HG	cg03830443	S-Shore	ZFPM2	cg22398226	N-Shore	
cg07547765	Island	HOXB7	cg24199558	Open Sea		cg02920129	Open Sea	ZNF385D
cg07061387	Open Sea	CD200R1	cg12628061	Open Sea		cg13254363	Open Sea	
cg24348981	Open Sea		cg13420364	Open Sea		cg05346619	Open Sea	
cg13329407	Open Sea		cg18647570	Open Sea	DHX8	cg10097598	Open Sea	
cg19567415	Open Sea	TANC2	cg19509778	Island	FOXI2	cg19076536	Open Sea	

**Fig. 5:**
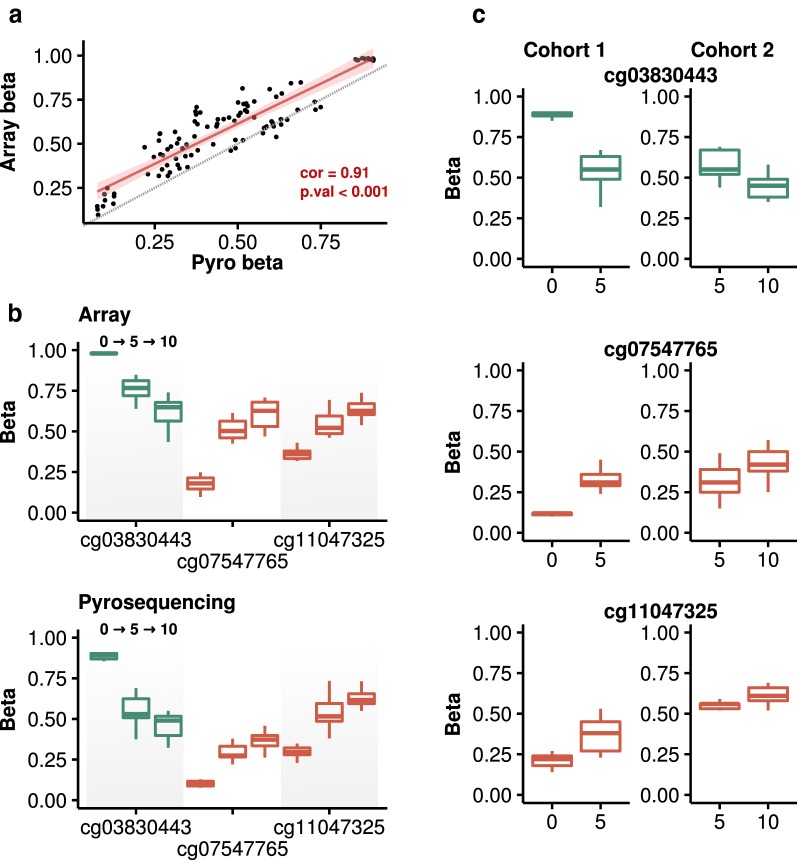
Technical and experimental validations. **a** Scatter plot showing the correlation between DNA methylation measurements performed by the Infinium MethylationEPIC BeadChip and by bisulfite pyrosequencing for 3 CpGs across the 11 subjects at the 3 time points (red, linear correlation estimation; grey, 1:1 line). **b** Box plots depicting the DNA methylation values of 3 CpGs measured by the Infinium MethylationEPIC BeadChip (top) and by bisulfite pyrosequencing (bottom). **c** Box plots indicating the DNA methylation values of the same 3 CpGs measured by bisulfite pyrosequencing in two independent cohorts, each comprising 9 children: measured (1) at 0 and 5 years of age and (2) at 5 and 10 years of age

Finally, we performed both technical and experimental bisulfite pyrosequencing validations of three of the CpGs found at the *HOXB7* (cg07547765), *SOCS3* (cg11047325) and *ZFPM2* (cg03830443) genes (Fig. 5, see Additional file 13: Table S7 for full data). Firstly, we analyzed the methylation value of the 11 subjects at three time points (n = 33), which demonstrated a very strong correlation between the Infinium MethylationEPIC BeadChip and pyrosequencing technology (Fig. 5a, b, Pearson correlation coefficient = 0.91, p < 0.001). Secondly, we evaluated the methylation status of the same CpGs in the peripheral blood of an independent longitudinal cohort of 9 children measured at birth and 5 years of age (n = 18), along with another independent longitudinal cohort of 9 different children measured at 5 and 10 years of age (n = 18) (Fig. 5c, see Additional file 1: Table S9 for the clinical information relating to these cohorts). This biological validation showed that the DNA methylation alterations identified can be reproduced in independent cohorts, thus underscoring the significance and robustness of the methylation changes found in this study.

## Discussion

In the present study we examined the genome-wide methylation profile of 33 longitudinal blood samples from 11 children at 0 (newborn), 5 and 10 years of age. Firstly, we found that extensive DNA methylation changes occur during the first 5 years of life, while much less epigenetic remodeling takes place in the following 5 years. As many as 110,726 CpG sites were found to have altered DNA methylation values when comparing newborn and 5 year-old samples, with a slight tendency toward an increase in methylation with age (54% of dmCpGs), while only 460 CpGs exhibited significant methylation changes between 5 and 10 years of age, 72% of which lost methylation. These findings are in line with the current literature which describes early-years epigenetic changes as being the most important of the postnatal period [8]. What is more, our three time point study design allowed us to directly compare the alterations occurring during two different early-years intervals, demonstrating that after 5 years, epigenetic reshaping is dramatically reduced. In the same vein, Acevedo and colleagues in their reporting of longitudinal DNA methylation changes during the first 5 years of life noted that in the third year these already seem to be weakening [19]. Aside from being more numerous, we found DNA methylation changes to be far more pronounced in the first lustrum of life than the subsequent one, which may have importance in terms of the functional influence of these methylation alterations on gene expression. Moreover, this observation did not seem to be related to considerable changes in cell-type composition occurring in the first 5 years as compared to the following 5.

We next examined the genomic distribution of the dmCpGs found, which revealed that hyper- and hypomethylation changes occur at very different locations: loss of methylation being observed at CpG-poor regions such as open sea locations, including intronic and intergenic regions, for both 0 → 5 and 5 → 10 changes, while methylation gain occurred at CpG-denser regions like CpG islands and gene promoters for both of the age intervals examined (although 0 → 5 changes were more similar to array distribution). These findings, with exceptions [19] are in line with most studies describing age-related DNA methylation changes, both in the early years [8, 16] and later years of aging [44, 45]. The fact that the great majority of hypomethylation changes were found at open sea regions underscores the value of screening technologies that examine CpG-sparse regions to accurately characterize DNA hypomethylation scenarios.

After identifying the genomic distribution of the age-related methylation changes, we sought to characterize the functional contexts associated with these loci. To achieve this, we integrated our DNA methylation data with external datasets describing histone modifications, chromatin states and enhancers for different tissue and cell types. These analyses primarily considered 0 → 5 dmCpGs because of the low number of 5 → 10 dmCpGs detected, which hindered the enrichments. The results evidenced a link between DNA hypermethylation and the repressive histone mark H3K27me3, and with chromatin states related to polycomb repressive domains and bivalent chromatin enhancers, while DNA hypomethylation occurred mainly at H3K4me1 regions and intragenic enhancers, as has been previously shown [46, 47]. Furthermore, when mapping the methylation changes to an enhancer library of 65 different tissues, we found that hypermethylation displayed a much lower overall enrichment at enhancer sites than hypomethylation, and while the former occurred mainly at enhancers in normal and embryonic tissue, the latter was particularly enriched in tissues and cell types related to blood. These observations suggest differences in the functionality of the enhancer-associated methylation changes that occur during the first 5 years of life depending on whether there is a gain or loss of methylation. Moreover, as studies are increasingly describing enhancer-associated DNA methylation as the main expression-correlated methylation phenomenon [20, 48, 49], our results point towards the enhancer-enriched DNA hypomethylation observed perhaps being of more importance in the control of gene expression than DNA hypermethylation. Indeed, enhancer methylation is in general negatively correlated to their activity [50], and thus it would make sense that the epigenetic changes that occur during the final stages of development are accompanied by the inactivation of general developmental enhancer elements, while tissue-specific (blood) enhancers become activated. In this setting, our data suggest that the early-life DNA methylation loss enriched at tissue-specific enhancer regions observed in our data could reflect the establishment of epigenetic active patterns defining tissue function during this period of life, while enhancers associated to development are already epigenetically repressed and suffer less important (hypermethylation) changes.

Another approach to studying the functional importance of DNA methylation changes is through the mapping of dmCpG sites to genes and then analyzing the related ontologies. Given that DNA methylation is irregularly distributed throughout the different parts of a gene [1], and because a great proportion of our mapped genes contained both hyper- and hypomethylated dmCpGs, we only performed the ontology analyses, firstly, on genes that exclusively either gained or lost methylation. We found that 0 → 5 DNA hypermethylation changes were principally related to developmental functions and cell-to-cell signaling, while hypomethylation was primarily linked to mRNA and protein metabolism, immune response and mitosis. It is worth mentioning that hypermethylation was also associated with terms such as growth, reproduction and response to retinoic acid, a molecule involved in organogenesis [51] as well as hematopoiesis [52]. Significantly, when we considered the ontologies of genes containing both hyper- and hypomethylated probes, we found considerable differences, implying that these genes could play different functional roles to those that are exclusively hyper- or hypomethylated. Moreover, when we examined the ontologies without making these distinctions, we found that, while hypermethylation ontologies did not considerably change, hypomethylation terms shifted from mRNA and protein metabolism to cellular localization and activation or GTPase activity (maintaining immunological terms). These latter findings are more in line with those reported in previous works which did not describe separating exclusively hyper- and hypomethylated genes [8, 14, 16, 19]. However, the fact that we found a change in function suggests that not taking into consideration the presence of concurrently hyper- and hypomethylated genes could affect the conclusions drawn from gene ontology analyses. On the whole, these results suggest that it is not only differentially hyper- or hypomethylated enhancers that have differing roles, but also differentially hyper- or hypomethylated genes, with the former being more related to general developmental functions, and the latter being involved in, among other things, immunological functions, thus reflecting the functionality differences observed for enhancer elements. In a final analysis, we further segregated the 0 → 5 dmCpGs into groups depending on their gene location (promoter, gene body, intron, exon) and also found changes between the gene ontologies of genes containing promoter- or exon-associated dmCpGs versus intronic- or gene body-associated dmCpGs, both for hyper- and hypomethylated CpGs. These observations imply that different genes, associated to different functions, suffer DNA methylation changes in different regions, which could help explain why DNA methylation changes in different contexts can have different consequences, and also suggests that the response of the gene elements to epigenetic changes during early life is gene region-specific.

Finally, we compared the DNA methylation changes that occur during the first two lustra of life, finding that the majority of the 5 → 10 changes are in fact a continuation of the 0 → 5 changes. By looking at the dmCpGs with the most substantial change in both age groups we defined 36 CpG sites with consistent DNA methylation changes during the first 10 years of life. These locations followed one of two trends: (1) strong 0 → 5 changes followed by weak 5 → 10 changes or (2) moderate overall 0 → 5→10 changes. Many of these CpGs were located at genes with important functions, such as development-associated *HOXB7* [53], which contained 2 CpGs, and the GATA-interacting *ZFPM*2 [54]. Interestingly, although both these genes are related to developmental functions, the first was found to be hypermethylated while the second was hypomethylated, and, what is more, the dmCpG associated with *ZFPM2* was also mapped to an enhancer element (Fig. 4c). Another zinc finger-family gene which we found to be altered, *ZNF385D*, has been associated to language impairment and reading disability in children [55]. Although this observation could be related to its function during prenatal brain development, the fact that the gene-associated dmCpG (cg02920129) shows a similar trend of methylation gain during both the 0 → 5 and 5 → 10 periods of life (Additional file 12: Figure S4) points towards a possible functional relevance throughout early life. It would thus be of interest to further study the link between the methylation status of *ZNF385D* and language impairment or reading disability. The *CD200R1* gene, which encodes for a myeloid- and T-cell distinctive transmembrane receptor [56] was found to be linked to an hypomethylated dmCpG (cg07061387), which was subsequently found to be associated with enhancer elements related to up to 3 different genes (*ATG3*, *NEPRO*, *GCSAM*) when the 0 → 5 → 10 dmCpGs were mapped to enhancers in different blood tissues, indicating that at least some of the DNA methylation changes found could exert an influence on genes other than the those on which the dmCpGs are located.

Lastly, we performed technical and biological validations of the methylation status of 3 dmCpGs located on the *HOXB7*, the *SOCS3* and the *ZFPM2* gene using bisulfite pyrosequencing. As expected, we observed a high correlation between the results of the Infinium MethylationEPIC BeadChip analysis and the pyrosequencing, albeit the DNA methylation values detected by the array were slightly higher, a fact which has been noted before [57]. Subsequently we examined the methylation of these CpGs in two independent longitudinal cohorts spanning the first and second lustra of life, and found that the DNA methylation changes found in the previous experiments were robust and reproducible in independent subjects.

## Conclusions

Collectively our results show that major epigenetic remodeling takes place during the first 5 years of life. However, despite a dramatic reduction, some genomic *loci* continue to experience considerable DNA methylation changes during the second lustrum of life. This observation illustrates that there are CpG sites whose change in methylation status could have functional roles beyond early postnatal development. In addition, we show that genes that experience simultaneous hyper- and hypomethylation are functionally distinct from those that are exclusively hyper- or hypomethylated. Finally, we have found that enhancer-associated methylation is different in the hyper- and hypomethylation scenario, the latter being more relevant and related to blood-specific enhancer elements, while developmental enhancer elements could already be epigenetically defined at birth, suffering changes of lesser importance during early life.

## Additional files

**Additional file 1: Table S9.** Clinical characteristics of the 18 subjects from the independent cohorts.

**Additional file 2: Table S8.** Primer sequences for bisulfite sequencing of the validated CpGs.

**Additional file 3: Table S1.** Lists of annotated 0→5 and 5→10 dmCpGs.

**Additional file 4: Table S6.** Gene ontology enrichment analysis of 0→5 and 5→10 dmCpGs. Enrichments were calculated from the difference between the dmCpGs obtained in each of the analyses and the GO ontology database by the R/Bioconductor package missMethyl. Ontologies for genes with 1) both hyper- and hypomethylated probes 2) exclusively either hyper- or hypomethyated and 3) from mapped hyper- or hypomethylated dmCpGs which did not take into account CpGs differentially methylated in the opposite direction in the same genes are included. Also included are the ontologies found when analyzing 0→5 dmCpGs grouped by gene region (promoter, exon, intron and gene body). All of the analyses include “molecular function”, “cellular component” and “biological process” terms.

**Additional file 5: Figure S1.** Boxplots indicating the distribution of absolute beta values of the DNA methylation changes for 0→5 and 5→10 hyper- and hypomethylated dmCpGs. Effect size is measured as median difference and Cliff’s delta.

**Additional file 6: Table S2.** Blood cell-type compositions as predicted by the Houseman algorithm for the 33 samples.

**Additional file 7: Table S3.** Histone mark enrichment analysis of 0→5 and 5→10 dmCpGs. Enrichments were calculated based on differences between the dmCpGs obtained in each of the analyses and the full collection of Roadmap epigenomics hg19 regions integrated in the LOLA extended software. Corresponding array backgrounds were used for the different comparisons.

**Additional file 8: Table S4.** Chromatin state enrichment analysis of 0→5 and 5→10 dmCpGs. Enrichments were calculated based on differences between the dmCpGs obtained in each of the analyses and the hg19 chromatin segmentation regions (ChromHMM, 18 states) obtained from Roadmap and ENCODE consortia. A custom LOLA database including information related to the chromatin states in the different tissues/cell lines and corresponding array backgrounds were used for the correct enrichment calculation.

**Additional file 9: Table S5.** Enhancer element enrichment analysis of 0→5 and 5→10 dmCpGs. Enrichments were calculated from the difference between the dmCpGs obtained in each of the analyses and the enhancer elements obtained from the EnhancerAtlas database. A customized LOLA database which included information related to the enhancers in the different tissues/cell lines and corresponding array backgrounds were used for the appropriate enrichment calculation.

**Additional file 10: Figure S2.** a) Treemap plots indicating the results of REViGO sematic analyses of significantly enriched (FDR < 0.05) gene ontology biological process terms for genes that simultaneously contained 0→5 hyper- and hypomethylated dmCpGs. In total, 460 significant terms were found (see Table S6 for full results, including Molecular Function and Cellular Component terms, and also 5→10 dmCpG enrichments). b) Equivalent plots for genes containing, respectively, hyper- or hypomethylated dmCpGs, irrespective of those same genes also containing dmCpGs that changed in the opposite direction.

**Additional file 11: Figure S3.** Treemap plots indicating the results of REViGO sematic analyses of significantly enriched (FDR < 0.05) gene ontology biological process terms for genes containing (a) hyper- and (b) hypomethylated 0→5 dmCpGs. The dmCpGs are grouped by annotated genomic location (see Table S1, column “annotation”, for the annotations; “promoter” is formed by collapsing “Distal promoter” and “Promoter (<= 1kb)”, “gene body” is formed by collapsing “3’ UTR”, “5’ UTR”, “Intron”, “Exon” and “Downstream”). See Table S6 for full results, including Molecular Function and Cellular Component terms.

**Additional file 12: Figure S4.** Boxplots showing the DNA methylation beta values of the 36 common 0→5→10 dmCpGs described in Table 2.

**Additional file 13: Table S7.** DNA methylation values obtained by bisulfite sequencing for 3 CpGs across the original 33 subjects (technical validation) and two independent longitudinal cohorts (biological validation).

## Abbreviations

ATG3: autophagy related 3
BF: breast feeding
BMIQ: beta-mixture quantile normalization
CD: Cliff’s delta
CS: cesarean section
dmCpGs: differentially methylated CpG
EHM: expected hypergeometric mean
ENCODE: encyclopedia of DNA elements
FDR: false discovery rate
FF: formula feeding
GCSAM: germinal center-associated signaling and motility protein
HDL: high-density lipoprotein
HMM: hidden Markov model
HOXB7: homeobox protein Hox-B7
IDAT: intensity data file
LDL: low-density lipoprotein
MD: median difference
MDS: multidimensional scaling
NEPRO: nucleolus and neural progenitor protein
NIH: National Institutes of Health
OR: odds ratio
PCA: principal component analysis
PCR: polymerase chain reaction
SGA/AGA/LGA: small/appropriate/large for gestational age
SOCS3: suppressor of cytokine signaling 3
VD: vaginal delivery
ZFPM2: zinc finger protein, FOG family member 2
ZNF385D: zinc finger protein 385D
