# Supplementary material for: Longitudinal genome-wide DNA methylation analysis uncovers persistent early-life DNA methylation changes
Source: J Transl Med. 2019 Jan 9;17:15. doi: 10.1186/s12967-018-1751-9 (PMC6327427; doi:10.1186/s12967-018-1751-9)
Supplement: Supplementary file 5 — Additional file 5: Figure S1. Boxplots indicating the distribution of absolute beta values of the DNA methylation changes for 0→5 and 5→10 hyper- and hypomethylated dmCpGs. Effect size is measured as median difference and Cliff’s delta. [file 12967_2018_1751_MOESM5_ESM.pdf]

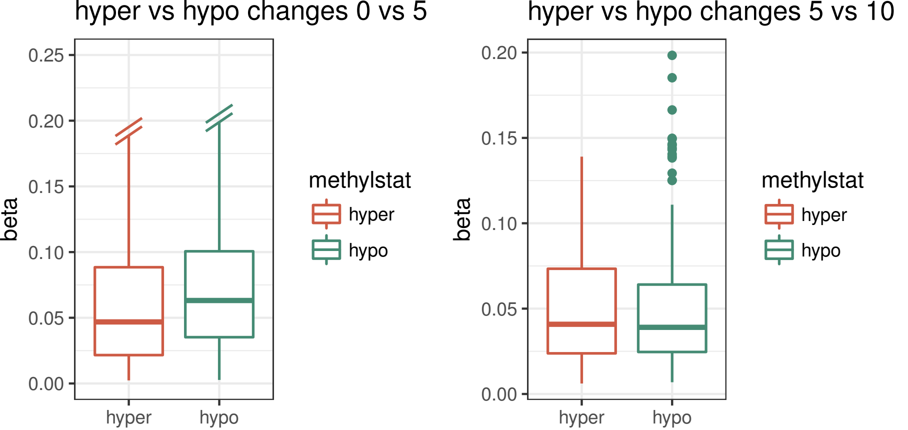

Wilcoxon test
p.val < 0.001
Cliff's delta = 0.2
Median difference = 0.02 (2%)

Wilcoxon test
p.val > 0.75 (non significant)
Cliff's delta = 0.02
Median difference = 0.002 (0.2%)
