# Supplementary figures and images for: Longitudinal genome-wide DNA methylation analysis uncovers persistent early-life DNA methylation changes
Source: J Transl Med. 2019 Jan 9;17:15. doi: 10.1186/s12967-018-1751-9 (PMC6327427; doi:10.1186/s12967-018-1751-9)

**both 0 vs 5**  
460 significant

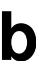

**hyper 0 vs 5**  
1184 significant

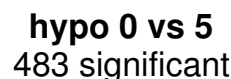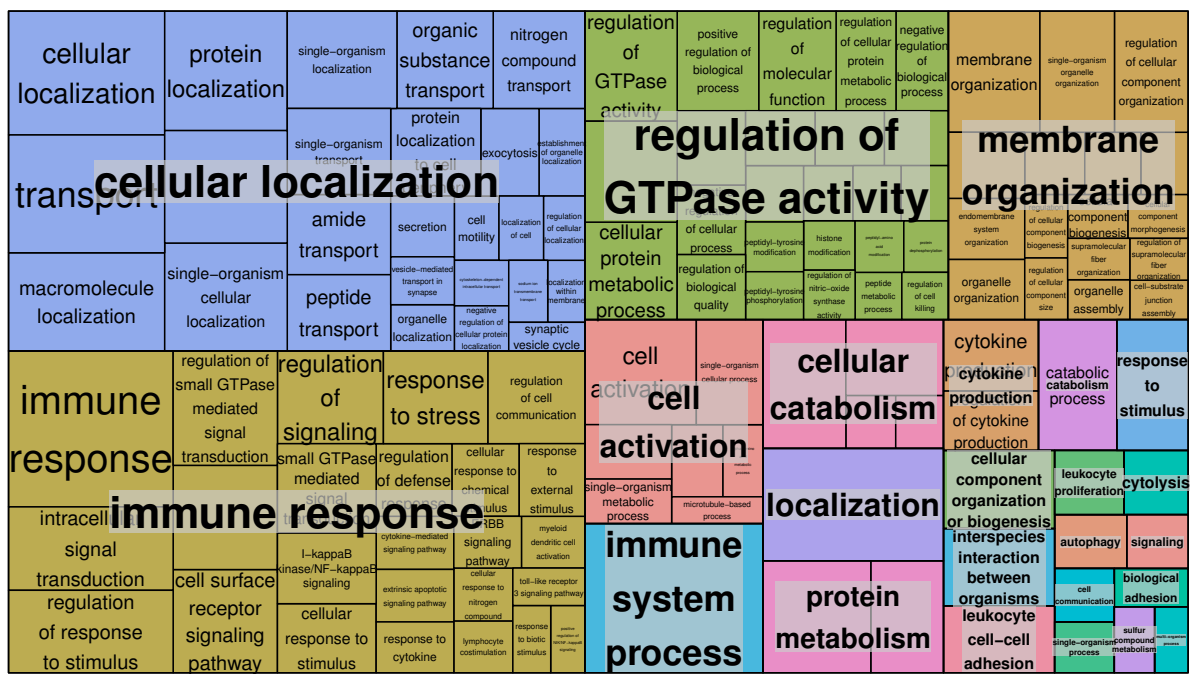

Supplement: Supplementary file 10 — Additional file 10: Figure S2. a) Treemap plots indicating the results of REViGO sematic analyses of significantly enriched (FDR < 0.05) gene ontology biological process terms for genes that simultaneously contained 0→5 hyper- and hypomethylated dmCpGs. In total, 460 significant terms were found (see Table S6 for full results, including Molecular Function and Cellular Component terms, and also 5→10 dmCpG enrichments). b) Equivalent plots for genes containing, respectively, hyper- or hypomethylated dmCpGs, irrespective of those same genes also containing dmCpGs that changed in the opposite direction. [file 12967_2018_1751_MOESM10_ESM.pdf]
