# Supplementary material for: Longitudinal genome-wide DNA methylation analysis uncovers persistent early-life DNA methylation changes
Source: J Transl Med. 2019 Jan 9;17:15. doi: 10.1186/s12967-018-1751-9 (PMC6327427; doi:10.1186/s12967-018-1751-9)
Supplement: Supplementary file 11 — Additional file 11: Figure S3. Treemap plots indicating the results of REViGO sematic analyses of significantly enriched (FDR < 0.05) gene ontology biological process terms for genes containing (a) hyper- and (b) hypomethylated 0→5 dmCpGs. The dmCpGs are grouped by annotated genomic location (see Table S1, column “annotation”, for the annotations; “promoter” is formed by collapsing “Distal promoter” and “Promoter (<= 1kb)”, “gene body” is formed by collapsing “3’ UTR”, “5’ UTR”, “Intron”, “Exon” and “Downstream”). See Table S6 for full results, including Molecular Function and Cellular Component terms. [file 12967_2018_1751_MOESM11_ESM.pdf]

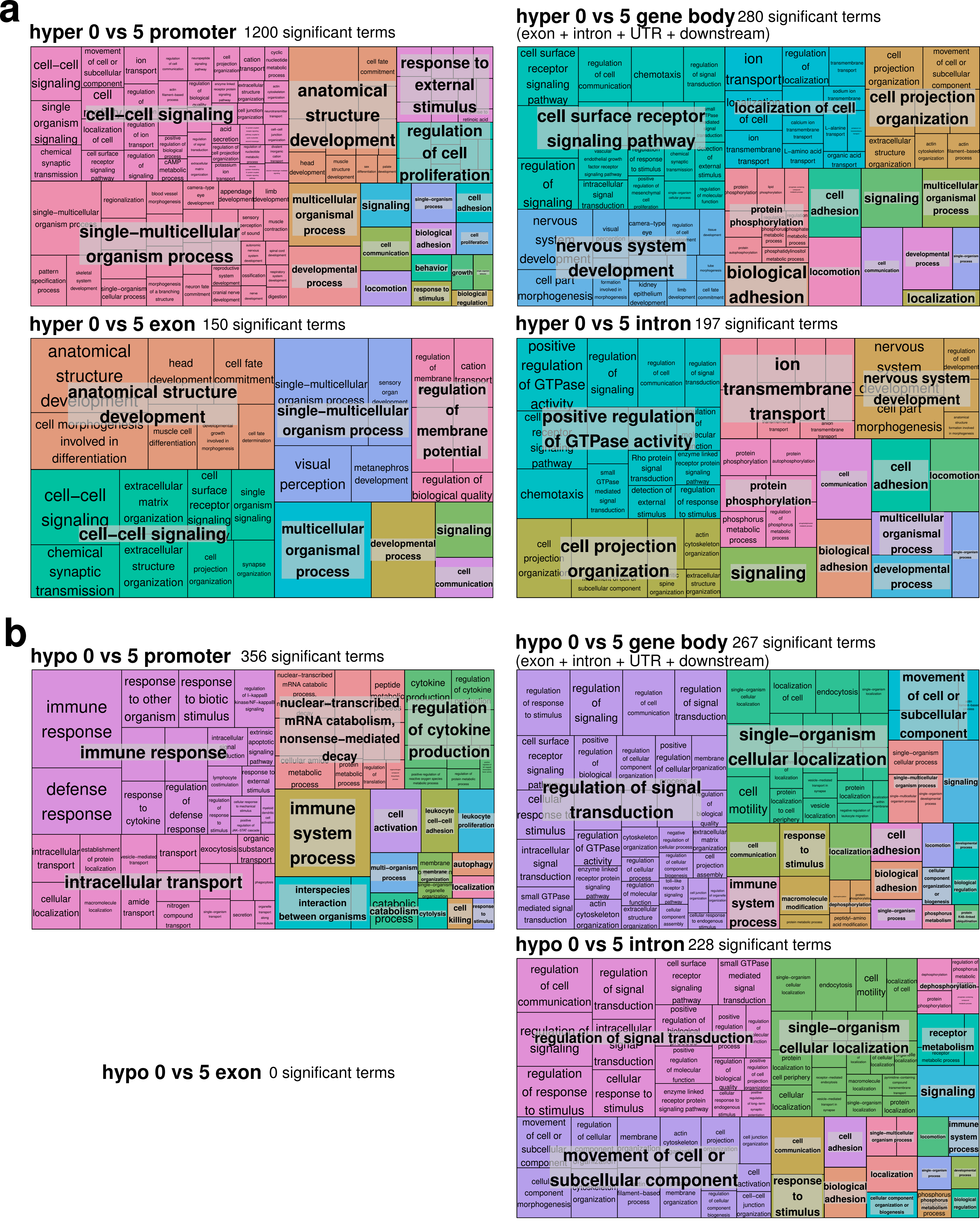

a
hyper 0 vs 5 promoter 1200 significant terms
cell–cell signaling
single–multicellular organism process
anatomical structure development
response to external stimulus
regulation of cell proliferation
multicellular organismal process
signaling
developmental process
biological adhesion
cell adhesion
cell communication
locomotion
behavior
growth
hyper 0 vs 5 gene body 280 significant terms
(exon + intron + UTR + downstream)
cell surface receptor signaling pathway
localization of cell
cell projection organization
nervous system development
biological adhesion
cell adhesion
protein phosphorylation
signaling
multicellular organismal process
developmental process
locomotion
localization
hyper 0 vs 5 exon 150 significant terms
anatomical structure development
single–multicellular organism process
regulation of membrane potential
cell–cell signaling
visual perception
multicellular organismal process
developmental process
signaling
cell communication
hyper 0 vs 5 intron 197 significant terms
ion transmembrane transport
nervous system development
positive regulation of GTPase activity
protein phosphorylation
cell adhesion
cell projection organization
signaling
multicellular organismal process
biological adhesion
developmental process
locomotion
b
hypo 0 vs 5 promoter 356 significant terms
immune response
nuclear–transcribed mRNA catabolism, nonsense–mediated decay
regulation of cytokine production
defense response
immune system process
intracellular transport
interspecies interaction between organisms
cell activation
leukocyte proliferation
autophagy
localization
catabolism
cell killing
hypo 0 vs 5 gene body 267 significant terms
(exon + intron + UTR + downstream)
movement of cell or subcellular component
single–organism cellular localization
regulation of signal transduction
cell motility
signaling
immune system process
response to stimulus
cell adhesion
biological adhesion
cell communication
hypo 0 vs 5 exon 0 significant terms
hypo 0 vs 5 intron 228 significant terms
regulation of signal transduction
single–organism cellular localization
receptor metabolism
signaling
movement of cell or subcellular component
response to stimulus
biological adhesion
cell adhesion
cell communication
localization
immune system process
