# Supplementary material for: Longitudinal genome-wide DNA methylation analysis uncovers persistent early-life DNA methylation changes
Source: J Transl Med. 2019 Jan 9;17:15. doi: 10.1186/s12967-018-1751-9 (PMC6327427; doi:10.1186/s12967-018-1751-9)
Supplement: Supplementary file 12 — Additional file 12: Figure S4. Boxplots showing the DNA methylation beta values of the 36 common 0→5→10 dmCpGs described in Table 2. [file 12967_2018_1751_MOESM12_ESM.pdf]

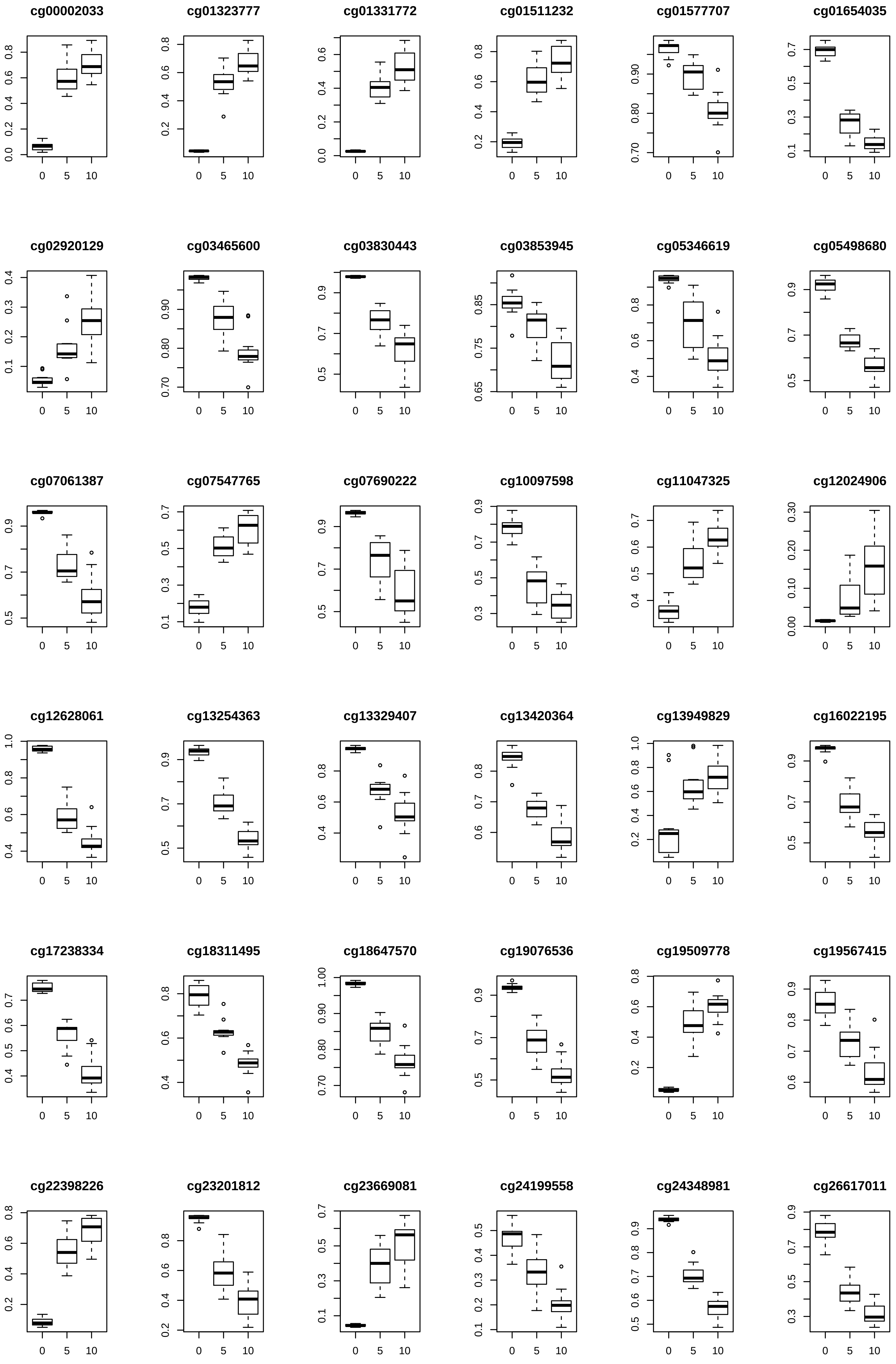
cg00002033
cg01323777
cg01331772
cg01511232
cg01577707
cg01654035
cg02920129
cg03465600
cg03830443
cg03853945
cg05346619
cg05498680
cg07061387
cg07547765
cg07690222
cg10097598
cg11047325
cg12024906
cg12628061
cg13254363
cg13329407
cg13420364
cg13949829
cg16022195
cg17238334
cg18311495
cg18647570
cg19076536
cg19509778
cg19567415
cg22398226
cg23201812
cg23669081
cg24199558
cg24348981
cg26617011
0
5
10
